# Knowledge of obstetric danger signs and associated factors among pregnant women in Erer district, Somali region, Ethiopia

**DOI:** 10.1186/s12905-016-0309-3

**Published:** 2016-06-06

**Authors:** Nebiyu Maseresha, Kifle Woldemichael, Lamessa Dube

**Affiliations:** 1 Erer District Health office, Ethiopia –Somali region, Somali, Ethiopia; 2 Department of Epidemiology, Jimma University, Jimma, Ethiopia

**Keywords:** Knowledge, Obstetric danger signs, Pregnant women, Erer district

## Abstract

**Background:**

Knowledge of danger signs of obstetric complications is first step in the appropriate and timely referral to essential obstetric care. Although women’s knowledge about the obstetric danger signs is important for improving maternal and child health, little is known about the current knowledge and influencing factors in pastoral community of Ethiopia. This study, therefore, aims to fill this gap by assessing the current level of knowledge and associated factors of pregnant women living in *Erer* district of Somali region, Ethiopia.

**Methods:**

A community based, cross-sectional study was conducted from April 7 to 21, 2014. The study involved 666 pregnant women residing in the district. Two-stage sampling technique was used to select the study subjects. Data about women’s socio-demographic information, reproductive history, knowledge of the danger signs, exposure to media and interventions were collected by interviewer administered questionnaires. A respondent who spontaneously mentioned at least two of the danger signs during each of the three periods was considered knowledgeable; otherwise not. Descriptive, bivariate, then multivariable logistic regression were done.

**Results:**

Six hundred thirty two pregnant women were interviewed with a response rate of 94.9 %. Only 98 (15.5 %) respondents were knowledgeable about obstetric danger signs. Urban residence [AOR = 2.43; 95 % CI (1.40, 4.21)], women who had been pregnant five or more times [AOR = 6.65; 95 % CI (2.48, 17.89)] and antenatal care utilization [AOR = 5.44; 95 % CI (3.26, 9.09)] were associated with being knowledgeable about obstetric danger signs during pregnancy, childbirth and postpartum.

**Conclusion:**

A significant proportion of pregnant women in Erer district do not have knowledge of obstetric danger signs. The implication is that lack of recognition may lead to delay in seeking care. Area of residence, gravidity and antenatal care service utilization are independently associated with the knowledge of women on obstetric danger signs in Erer district, a pastoralist community. Thus, intervention programs aiming to improve women’s knowledge about obstetric danger signs and symptoms should consider the factors independently associated.

**Electronic supplementary material:**

The online version of this article (doi:10.1186/s12905-016-0309-3) contains supplementary material, which is available to authorized users.

## Background

Maternal mortality refers to deaths due to complications from pregnancy or childbirth. According to UN inter-agency estimates the global maternal mortality ratio declined by 45 % from 1990 to 2013. The number of women and girls who died each year from complications of pregnancy and childbirth declined from 523,000 in 1990 to 289,000 in 2013. While impressive, this is less than half the rate needed to achieve the three-quarters reduction in maternal mortality targeted for 2015 in Millennium Development Goal 5. Still, about 800 women are dying each day in the process of giving life. Almost all maternal deaths (99 %) occur in developing countries [1, 2]. The level of maternal mortality in Ethiopia is among the highest in the world. According to the Ethiopian demography and health survey (EDHS) 2011 report, maternal mortality ratio is 676 per 100,000 live births [3].

About 72 % of maternal deaths are attributed to direct causes of maternal mortality [2, 4], including hemorrhage 27 %, hypertension 14 %, sepsis 11 %, abortion 8 %,embolism 3 % and other direct causes [2]. While most pregnancies and births are uneventful, some pregnancies are at risk. Around 15 % of all pregnant women develop a potentially life-threatening complication that calls for skilled care and some require a major obstetrical intervention to survive [5, 6]. Most of these life-threatening complications are unpredictable. However, they are preventable if timely decisions are made to access quality emergency obstetric care services. Thus, reduction of maternal mortality hinges on minimizing the delays in getting appropriate emergency care at times of complications [7, 8].

Obstetric danger signs are complications that the woman and her family can easily recognize, and they are different depending on the obstetric period in which they occur. The most common signs are severe vaginal bleeding, swollen hands/face and blurred vision during pregnancy; severe vaginal bleeding, prolonged labor, convulsions and retained placenta during labor and childbirth and severe vaginal bleeding, foul-smelling vaginal discharge, and fever during the postpartum period [9–11].

If women and their families can recognize the obstetric danger signs and promptly seek health care services, significant amount of maternal morbidity and mortality could be prevented. Therefore, increasing women’s knowledge about the obstetric danger signs would improve early detection of problems and reduce the delay in deciding to seek obstetric care [12, 13]. Thus, one of the key strategies for reducing maternal mortality is increasing knowledge of the obstetric danger signs among women, family and community at large [14].

Increasing knowledge of obstetric danger signs and birth preparedness are strategies aimed at enhancing the utilization of skilled care during low-risk births and emergency obstetric care in complicated cases in low income countries [10, 15, 16]. As in many developing countries, knowledge of danger signs remains low in Ethiopia [17–23]. Cross-sectional studies done in agrarian communities and urban dwellers in different regions of Ethiopia consistently reported that women’s knowledge about obstetric danger signs is low. The proportion of women who had knowledge about the danger signs of obstetric complication ranges from 24.1 % to 61.1 % [20–23]. To improve awareness of danger signs and symptoms during pregnancy, labor, delivery, and post-partum, the national reproductive strategy of Ethiopia had included a strategy to ensure 80% of all households/families recognize at least three danger signs associated with pregnancy-related complications in areas where Health Extension Programs are fully implemented by year 2010 [15]. Health Extension Program (HEP) is an innovative community based health care delivery system aimed at providing essential preventive health care services. It was introduced in recognition of failure of essential services to reach communities in remote parts of Ethiopia. HEP services have been customized to meet the needs, demands and expectations of the pastoralist, agrarian and urban population. It, therefore, introduced a new cadre of health worker, Health Extension Workers (HEWs), and defined a package of essential interventions for them to deliver from village health posts [24]. A recent report of Ethiopian Federal Ministry of Health showed a wide variation in ANC coverage across regions, ranging from the lowest 41.6 % in Somali to the highest 100 % in Tigray region. About 80 % of the ANC clients were not told about pregnancy complications [25].

Although increasing women’s knowledge about obstetric danger signs is important for improving maternal and child health, little is known about the current knowledge and influencing factors in pastoralist community, like Somali region, one of the nine regions in Ethiopia. Based on 2007 figures from the Central Statistical Agency of Ethiopia, the Somali Region has an estimated total population of 4,713,619 consisting of 2,621,430 men and 2,092,189 women. Eighty six percent of the population is estimated to be rural inhabitants, while 13.9 % are urban dwellers [26]. Pastoralism is the most common livelihood, comprising about 60 % of the region’s rural population followed by Agropastoralism comprising about 25 % of the total rural population [27]. According to the Health and Health Related Indicators published in 2014 by Ministry of health, currently there are 10 hospitals, 140 health centers and 1062 Health Posts in the region, making 102.8 % primary health coverage of the region [28].

This study aims to fill this gap by assessing the current level of women’s knowledge and associated factors of obstetric danger signs among pregnant women living in Erer district of Somali region, Ethiopia. Therefore, the finding of this study is important to guide public health planners and implementers in planning and designing appropriate interventions strategies in order to increase women’s knowledge on obstetric danger signs.

## Methods

Erer district is one of the seven districts in Shinile administrative zone of Ethiopia’s Somali region. It is located in the eastern part of Ethiopia, 563 Km from Addis Ababa (the capital city of Ethiopia). The district is divided into fourteen (13 rural, 1 urban) kebeles (the smallest administrative unit) with a total population of 92,807 as estimated from census 2007 and 75–80 % is pastoral livelihood.

A community based, cross–sectional study design was employed to assess knowledge of obstetric danger signs and associated factors among pregnant women. The study was conducted from April 7 to 21, 2014. The source population was all pregnant women living in the Erer district during the study period. Randomly selected pregnant women who were living in the district during the study period were included in the study.

A sample size of 660 was determined using single population proportion formula [29].

$$ n=\frac{{\displaystyle {z}_a^2}p\left(1-P\right)}{d^2} $$

With the following assumptions: proportion(*p*) of women who mention at least two danger signs of pregnancy to be 58.8 % as estimated from other study [23], confidence interval 95 %, degree of precision(*d*),5 % and design effect, 2. Then, finite population correction was used because the expected number of pregnant women in the district was 3115 that is less than 10,000 and finally adding 10 % non-response rate.

A two-stage stratified sampling technique was used to select study subjects. In the first stage, a list of kebeles was stratified into urban and rural. Eight out of 14 kebeles were ultimately selected. The urban kebele was selected purposively and seven rural kebeles (Gota, Kenteras, Billa, Germam, Hurso, Aydora and Asbulli) were selected randomly by lottery method. Then, in each selected kebele, a preliminary survey was done to register eligible pregnant women. The registration was conducted by the health extension workers and community health workers in collaboration with the district health office using a form that has serial number, name of the pregnant woman, her village and kebele names and house number if available. Then two sampling frames (one for the urban and one for the rural kebeles) were prepared by compiling the collected forms. The total sample size was allocated proportionally to both urban (136) and rural (524) kebeles based upon the number of pregnant women in each sampling frame. Finally, computer based simple random sampling method was used to select the study subjects. Subsequently, the lists of selected pregnant women with their address were distributed to the data collectors.

Data were collected through face-to-face interviews with pregnant women at their homes. A structured questionnaire (Additional file 1) was adapted from a safe motherhood questionnaire developed by the Maternal Neonatal Program of JHPIEGO, an affiliate of John Hopkins University (1). It contained four sections namely; socio-demographic information, reproductive history, knowledge of danger sign and exposure to media and interventions. The questionnaires were translated to Somali language. It was pre-tested in Dimtu kebele, which was not included in the main study.

Eight female diploma nurses, who can speak the local language (Somali), were recruited from the district as data collectors. Their knowledge of the study area (kebeles) and previous data collection experience was used as criteria for selection. Two health officers were recruited as supervisors for the study. The recruitment of data collectors and supervisors was done in collaboration with the district health office.

After the selection, both the data collectors and supervisors were trained for three days before the actual work. The training focused on the aim of study, study procedures and data collection techniques. During data collection all filled questionnaires were checked for completeness and consistency by the field supervisors.

Educational status of female participants and their partners were assessed independently. Additionally, respondents were asked if other household members had formal education. The knowledge of respondents was assessed by asking the respondents to mention the danger signs that can happen during pregnancy, childbirth and after childbirth. The question posed to participants to elicit responses on knowledge of key danger signs during the three phases was “In your opinion, what are some serious health problems that can occur during pregnancy, delivery and after delivery (postpartum) that could endanger the life of a woman? What else?” Only spontaneous responses were recorded. A respondent that answered at least two of the danger signs during each of the three phases (pregnancy, childbirth or postpartum) was coded knowledgeable; otherwise not [21]. Travel from health facilities was coded near for ‘≤30 min’ and far for ‘>30 min’. Gravida is number of pregnancies a woman ever has including current pregnancy and categorized in to three: ‘primigravida’, ‘multigravida 2–4’ and ‘gravida 5 or above’. A model family is defined by a household that has adopted most if not all of the government’s 16 priority interventions –from vaccinating their children and sleeping under mosquito bed nets to building separate latrines and using family planning and is categorized as ‘model family’ if the family has a certificate, otherwise they were not considered to be not a model family. The data were entered into EpiData version 3.1 and exported to SPSS for windows version 16 for analysis. Analysis was done sequentially starting with univariate using descriptive techniques, then bivariate analysis and finally multivariable logistic regression analysis to control for possible confounders. All independent variables found to be associated at *p* < 0.25 during the bivariate analyses were entered to the multivariable binary logistic regression for modeling. Statistical significance was declared at *p*-value less than 0.05. Multicollinearity between the independent variables was checked and gravidity, parity and live birth were detected as collinear and only gravidity was entered into the multivariable logistic analysis.

## Results

### Socio-demographic characteristics of the respondents

A total of 632 pregnant women were included in the study with a response rate of 95.8 %. The mean (±standard deviation (SD)) age of respondents was 25.7 (±4.3) years and the mean (±SD) family size of the respondents was 4.6 (±1.5) individuals. Majority of the respondents were Somali (92.2 %), Muslims (96.2 %) and married (97.9 %). Six hundred twelve (96.8 %) respondents were housewives. Five hundred ninety-nine (94.8 %) respondents did not receive formal education. Their husbands’ mean (±SD) age was 32.4(±5.9) years, 563 (91.0 %) of respondents’ husbands didn’t receive formal education, and 287 (46.4 %) were involved in cattle raising. Two hundred five (32.4 %) respondents lived with individuals who are currently attending formal education. Of these, 167 (83.1 %) completed elementary school and 34 (16.9 %) completed at least 9th grade. Either radio or television or both was available in 500 (79.1 %) respondents’ homes. Three hundred thirty one (52.4 %) and 221(35.0 %) of respondents claimed that they could reach health post and health center within 30 min on foot respectively (Table 1).

**Table 1:** Socio-demographic characteristics of respondents, Erer district, Somali region, Ethiopia, 2014 (*n* = 632)

Characteristics	Number (%)
Place of residence	
Urban	128 (20.3)
Rural	504 (79.7)
Age of respondents (in years)	
<20	44 (7)
20–29	488 (77.2)
≥30	100 (15.8)
Religion	
Muslim	608 (96.2)
Christian	24 (3.8)
Ethnicity	
Somali	583 (92.2)
Oromo	30 (4.7)
Others	19 (3.1)
Marital Status	
Married	619 (97.9)
Not married	13 (2.1)
Educational status	
Not attend formal education	599 (94.8)
Attends formal education	33 (5.2)
Occupation	
House wife	612 (96.8)
Employed	11 (1.8)
Merchant or Daily labourer	9 (1.4)
Husband’s educational status	
Not attend formal Education	563 (91.0)
Attends formal education	56 (9.0)
Husband’s occupation	
Cattle rearing	287 (46.4)
Farmer	198 (32.0)
Employed	42 (6.8)
Daily laborer	45 (7.3)
Merchant	36 (5.8)
Others	11 (1.7)
Family size	
<4	129 (20.4)
4–6	432 (68.4)
≥7	71 (11.2)
Having radio or Television	
Yes	500 (79.1)
No	132 (20.9)
Travel time to health center (in minutes)	
≤30	221 (35.0)
Travel time to health post (in minutes)	
≤30	331 (52.4)
Living with any family members currently attending formal education	
Yes	205 (32.4)

### Obstetric characteristics’ of respondents

Ninety two (14.6 %) respondents were pregnant for the first time and 130 (20.6 %) had been pregnant five or more times. From the total participants, 88 (13.9 %) had been visited by HEWs on door-to-door service provision during the current pregnancy. From this, 62 (70.5 %) and 26 (29.5 %) of respondents were visited once, and more than once, respectively. During the current pregnancy, 184 (29.1 %) of respondents had visited health facilities for ANC service. Of these, 106 (56.7 %) received the service from health posts. From the total respondents, 252 (39.9 %) reported that they had received health education on maternal health during the last year prior to data collection (Table 2).

**Table 2:** Obstetric characteristics of respondents in Erer district, Somali region, Ethiopia, 2014 (*n* = 632)

Variables	Number (%)
Gravidity	
1	92 (14.6)
2–4	410 (64.8)
5+	130 (20.6)
History of stillbirth	
Yes	19 (3.0)
No	613 (97.0)
Gestation age	
First trimester	108 (17.1)
2nd trimester	325 (51.4)
3rd trimester	199 (31.5)
Visited by Health extension workers	
Yes	88 (13.9)
No	544 (86.1)
Model family	
Yes	213 (33.7)
No	419 (66.3)
Antenatal care utilization	
Yes	187 (29.1)
No	445 (70.9)
Place of Antenatal care visit (*n* = 187)	
Health center	81 (43.3)
Health Post	106 (56.7)
Have received maternal health education	
Yes	252 (39.9)
No	380 (60.1)

### Knowledge of obstetric danger signs

Only 98 (15.5 %) respondents were knowledgeable about obstetric danger signs in all categories (pregnancy, childbirth and after childbirth). Around one third (31.8 %), one fourth (25.5 %) and around one fifth (19.1 %) of respondents had mentioned at least two danger sign during labor, pregnancy and postnatal period respectively.

When asked to mention danger signs during pregnancy, the most common spontaneously mentioned danger sign was ‘vaginal bleeding’. The commonly mentioned danger signs during labor and child birth include ‘prolonged labor’ and ‘excessive bleeding’. The most commonly mentioned danger signs of postpartum were ‘excessive bleeding’, ‘abdominal pain’ and ‘fever (Table 3).

**Table 3:** Obstetric danger signs as reported by the pregnant women, Erer district, Somali region, Ethiopia, 2014 (*n* = 632)

Danger signs	Pregnancy	Labor/delivery	Postpartum
	Number (%)	Number (%)	Number (%)
Vaginal bleeding	155 (0.25)	NA	125 (0.20)
Abdominal pain	44 (0.07)	14 (0.02)	62 (0.10)
Weakness	99 (0.16)	50 (0.08)	43 (0.07)
Reduced fetal movement	86 (0.14)	NA	NA
Swelling of hands, faces &legs	78 (0.12)	NA	NA
Blurred vision	48 (0.08)	NA	25 (0.04)
Breathing difficulty	39 (0.06)	NA	31 (0.05)
Severe headache	32 (0.05)	37 (0.06)	37 (0.06)
Fever	30 (0.05)	33 (0.05)	51 (0.08)
Convulsion	22 (0.03)	52 (0.08)	22 (0.03)
Labor more than 12 h	NA	162 (0.26)	NA
Abnormal fetal position	NA	100 (0.16)	NA
Excessive bleeding	NA	92 (0.15)	NA
Placenta not delivered within ½ hour	NA	52 (0.08)	NA
Foul smelling lochia	NA	NA	48 (0.08)
Loss of consciousness	NA	69 (0.11)	NA

*NA* not applicable

### Factors independently associated with the knowledge of obstetric danger signs

After adjusted for age, educational status, occupation and living with any family member currently attending formal education, pregnant women living in urban areas were 2.43 times more likely to have knowledge about obstetric danger signs compared to those living in rural areas [AOR = 2.43; 95 % CI (1.40, 4.21)]. Gravidity was another factor which was independently associated with the knowledge of obstetric danger signs. Women who had been pregnant five or more times were 6.65 times more likely to be knowledgeable about obstetric danger signs compared with primi-gravida women [AOR = 6.65; 95 % CI (2.48, 17.89)]. In addition, utilizing ANC services was independently associated with knowledge of danger signs. Pregnant women who utilized ANC services were 5.44 times more likely to be knowledgeable about the obstetric danger signs compared with those who didn’t [AOR = 5.44; 95 % CI (3.26, 9.10)] (Table 4).

**Table 4:** Bivariate and Multivariable analysis of Knowledge about obstetric danger signs with selected variables among respondents in Erer district, Somali region, Ethiopia, 2014 (*n* = 632)

Variables	Knowledge of ODS		Crude OR (95 % CI)	AOR (95 % CI)
	Yes	No		
Age (years)				
<20	42	2	Ref.	
20–29	415	73	3.694 (0.88,15.60)	
≥30	77	23	6.273 (1.41,27.90)	
Educational status				
Illiterate	519	80	Ref.	
Primary education	12	6	3.24 (1.18,8.89)	
secondary and above	3	12	25.95 (7.12, 93.98)	
Occupation				
Employed	4	7	Ref.	
Not employed	530	91	10.19 (2.93,35.52)	
Living with any family members currently attending formal education				
Yes	158	47	2.19 (1.15,3.40)	
No	376	51	Ref.	
Residence				
Urban	84	44	4.37 (2.75,6.92)	2.43 (1.40, 4.21) ^a^
Rural	450	54	Ref.	
Gravidity				
1	83	9	Ref.	
2–4	361	49	1.25 (0.59,2.65)	2.16 (0.85, 5.48)
≥5	90	40	4.10 (1.88,8.96)	6.65 (2.48, 17.89) ^a^
Antenatal care utilization				
Yes	120	67	7.46 (4.65,11.95)	5.44 (3.26,9.10) ^a^
No	414	31	Ref.	

*ODS* obstetric danger signs

^a^ significantly associated

## Discussion

This study showed that women’s knowledge of the danger signs of obstetric complications during pregnancy, childbirth and after childbirth was 15.5 % and factors associated with this knowledge were place of residence, gravidity and ANC care follow up. Women’s knowledge in this study is consistently lower than the cross-sectional studies done in Ethiopia: Arba Minch (24.1 %) and Aletawondo (30.9 %), South Nations Nationalities and Peoples Regional State (SNNPS) [20, 21]; Debre Birhan (38.6 %), Amhara Region [22]; and Tsegedie district (49.5 %), Tigray Region [23]. This difference might be attributed to socio-cultural differences as this study was conducted in pastoralist community in contrast to the previous studies set in agrarian or urban communities. Implementation of health intervention programs may also explain these differences.

The major causes of maternal mortality are hemorrhage, sepsis, and pregnancy induced hypertension. Pregnant mothers need to have adequate knowledge about the signs indicating these problems [15]. The fact that a large proportion of pregnant women did not know danger signs of serious health problems could adversely affect their preparedness for pregnancy complications.

Urban residence was found to have a significant association with being knowledgeable about obstetric danger signs during pregnancy, childbirth and postpartum period. This agrees with a study conducted in Southern Ethiopia and Debre Birhan [20, 22]. This could be due to the fact that urban residents have better access to health information and maternal health services as compared with rural counterparts.

Gravidity was another factor, which is independently associated with the knowledge of obstetric danger signs during pregnancy, childbirth and after childbirth. The same finding was reported from the study conducted in Albeheria [19] and Aleta Wendo [21]. This may be due to the fact women who experiences a previous pregnancy were more likely to differentiate abnormalities and might have learned from their experience.

ANC service utilization was found to have a significant association with the knowledge of obstetric danger signs during pregnancy, childbirth and postpartum period, and this observation is consistent with reports from Debre Birhan [22]. Antenatal care provides an opportunity to counsel women about possible serious danger signs of pregnancy.

The present study illustrates the current level of knowledge on obstetric danger signs and factors among pregnant women living in the Erer district of Somali region, Ethiopia. However, there are some limitations. First, pregnancy status was based on mothers’ self-report and pregnancy test was not applied. This may have introduced selection bias by excluding pregnant women who did not know their pregnancy status. Second, household wealth was not assessed. Finally, this cross-sectional design does not permit the examination of potential temporal relationships.

## Conclusions

A significant proportion of pregnant women in Erer district are not knowledgeable of obstetric danger signs during pregnancy, childbirth and postpartum. The implication is that lack of recognition may lead to delay in seeking care. Factors associated in Erer district– a pastoralist community are similar to the agrarian community. Area of residence, gravidity and ANC service utilization were independently associated with the knowledge of women on obstetric danger signs during the three periods. Thus, any intervention program aiming to reducing the maternal mortality should focus on women living pastoralist community like Erer district, in particular rural dwellers and lower parity women. Increasing ANC service utilization would improve pregnant women’s knowledge about obstetric danger signs and symptoms.

## Abbreviations

ANC, antenatal care; AOR, adjusted odds ratio; JHPIEGO, Johns Hopkins Program for International Education in Gynecology and Obstetrics; EDHS, Ethiopian Demography and Health Survey; HEP, health extension program; HEWs, health extension workers; SD, standard deviation; SNNPS, South Nations Nationalities and Peoples Regional State

## Additional file

Additional file 1: Questionnaire. (DOCX 56 kb)
