# Supplementary material for: Knowledge of obstetric danger signs and associated factors among pregnant women in Erer district, Somali region, Ethiopia
Source: BMC Womens Health. 2016 Jun 6;16:30. doi: 10.1186/s12905-016-0309-3 (PMC4893837; doi:10.1186/s12905-016-0309-3)
Supplement: Additional file 1: — Questionnaire. (DOCX 56 kb) [file 12905_2016_309_MOESM1_ESM.docx]

| **s/no.**  **Additional file 1: questionnaire** | **questions** | **Responses** | **Skip to** |
| --- | --- | --- | --- |
|  | **Socio-demographic Information’s** | |  |
| 1 | How old are you? | __________age in years |  |
| 2 | What is your religion? | 1. Muslims 2. Orthodox 3. Protestant 4. Other (specify)________ |  |
| 3 | What is your current marital Status? | 1. married 2. widowed 3. divorce 4. Other (specify)------------------ |  |
| 4 | To which ethnic group do you Belong to? | 1. Somali 2. Oromo 3. Amhara 4. Other, specify_________ |  |
| 5 | What is your occupation? | 1. Farmer 2. House wife 3. Cattle raring 4. Other, specify-____ |  |
| 6 | What is your monthly income? | ________(in Ethiopian Birr) |  |
| 7 | What is your educational  Status? | 1. Can’t read and write  2. Can read and write  3. Grade completed _______ |  |
| 8 | What is your Husband’s age? | __________age in years |  |
| 9 | What is your husband educational  Status? | 1. Can’t read and write  2. Can read and write  3. Formal education, Grade completed _______ |  |
| 10 | What is your husband occupation? | 1. Government worker 2. Farmer 3. Other (specify)_____ |  |
| 11 | Family size | ________ |  |
| 12 | Do you have any family members who attend formal education? | 1. Yes  2. No | If no. skip to Q14 |
| 13 | If yes, level of education | 1. Grade________ |  |
| 14 | Village | 1. Urban 2. Rural |  |
| 15 | How far is the nearest health facility from your house? | --------------minutes (on foot)  _____________ minutes (by mule) |  |
| 15a | How far is the nearest Health Post from your house? | --------------minutes (on foot)  _________ minutes (by mule) |  |
| 15b | How far is the nearest health center from your house? | --------------minutes (on foot)  _____________ minutes (by mule) |  |
| 16 | What is the nearest health facility? | 1. Health Post 2. Health Center 3. Hospital 4. Others(specify)_______ |  |
| 17 | Within last six months have you moved to other places from this kebele? | 1. Yes  2. No | If no , skip to Q19 |
| 18 | Why were you moving from present place? | 1. For search of grass and water for cattle  2. To ask relatives and other families  3. Other (specify)s_________ |  |
| 19 | Is your Household a Model Household? | 1. Yes 2. No |  |
| 20 | Do you have access for mass media? | 1. Yes 2. No |  |
| 21 | If yes, what do you use? | 1. Radio 2. TV 3. Newspaper 4. Other (specify)s______ |  |
|  | **Previous history of obstetric** | |  |
| 22 | Age at first marriage | _____________ |  |
| 23 | How many times in total you became pregnant? | _________ |  |
| 24 | How many times in total you gave birth? | ______________ |  |
| 25 | How many of your pregnancies resulted in a baby that was born Alive? | ___________ |  |
| 26 | How many of your pregnancies resulted in a baby that was born Dead? | ___________ |  |
| 27 | Do you have history of abortion? | 1.yes 2, no |  |
| 28 | If yes how many times? | _______ |  |
| 29 | Was there any complication /health problem during the previous pregnancy? | 1. Yes 2. No |  |
|  | **Current pregnancy /health service utilization** | |  |
| 30 | LMP /pregnancy in months | ______ |  |
| 31 | Does health extension visit  During this pregnancy? | 1. Yes 2. No | If no, skip to Q35 |
| 32 | If yes for Q (31), how many times? | _________ |  |
| 33 | Does the HEW give you any message about pregnancy? |  |  |
| 34 | What about it | ________________________ |  |
| 35 | Did you go to a health facility during this pregnancy? | 1. Yes 2. No | If no, skip to Q38 |
| 36 | If yes, where did you go? | 1. HC 2. HP   3-other____ |  |
| 37 | If yes, why did you go? | 1. For check up 2. I am sick 3. Other (specify)s______ |  |
| 38 | Do you ever have health education on maternal health? | 1. Yes 2. No | If no, skip to Q40 |
| 39 | If yes , by whom | 1. CHW  2. HEWs  3. Health professionals |  |
|  | **Knowledge about obstetric danger signs** | |  |
| 40 | Have you heard about any complications that can happen with pregnancy? | 1. Yes 2. No | If no, skip to Q42 |
| 41 | If yes, from whom have you heard? | 1. HEW 3- CHW 2. TBAs 4- HP |  |
| 42 | Do you know the danger signs of health problems during pregnancy? | 1. Yes 2. No | If no, skip to Q45 |
| 43 | If yes, can you mention them? (Mark x by asking what else do you know) | 1. vaginal bleeding 2. Convulsions 3. severe headaches 4. blurred vision 5. severe abdominal pain 6. Difficult breathing 7. High fever 8. Swelling of faces, legs. 9. Persistent vomiting 10. No fetal movements   88.Others specify------------- |  |
| 44 | Do you know the danger signs of health problems during labor and delivery? | 1. Yes 2. No | If no ,  Skip toQ46 |
| 45 | If yes can you mention them? | 1. Severe headache 2. Labor more than 12 hr 3. Heavy bleeding 4. High fever 5. Retained placenta 6. Vaginal bleeding 7. Abnormal fetal position   88.Others specify------------- |  |
| 46 | Do you know the danger signs of health problems during pregnancy? | 1. Yes 2. No |  |
| 47 | Do you know the danger signs of health problems after childbirth? | 1. Vaginal bleeding 2. Convulsions 3. Fast or difficult breathing 4. High fever 5. Too weak to get out of bed 6. Abdominal pain 7. Abdominal pain 8. Breasts swollen red or tender breasts, or sore 9. Foul-smelling lochia 10. Blurred Vision 11. Other (specify)s_________ |  |
| 48 | Where do you go if these danger signs happen? | 1. Health Centers 2. Hospital 3. Traditional birth attendants 4. Traditional healers 5. Other(specify)_____________ |  |
